# P-type ATPase zinc transporter Rv3270 of *Mycobacterium tuberculosis* enhances multi-drug efflux activity

**DOI:** 10.1099/mic.0.001441

**Published:** 2024-02-19

**Authors:** Debasmita Chatterjee, Aditya Prasad Panda, A. R. Daya Manasi, Anindya S. Ghosh

**Affiliations:** ^1^​ Department of Bioscience and Biotechnology, Indian Institute of Technology Kharagpur, Kharagpur, West Bengal 721302, India

**Keywords:** biofilm, efflux pump inhibitors, efflux pumps, metal–antibiotic cross resistance, metal transporter, *Mycobacterium tuberculosis*

## Abstract

Metal homeostasis is maintained by the uptake, storage and efflux of metal ions that are necessary for the survival of the bacterium. Homeostasis is mostly regulated by a group of transporters categorized as ABC transporters and P-type ATPases. On the other hand, efflux pumps often play a role in drug–metal cross-resistance. Here, with the help of antibiotic sensitivity, antibiotic/dye accumulation and semi-quantitative biofilm formation assessments we report the ability of Rv3270, a P-type ATPase known for its role in combating Mn^2+^ and Zn^2+^ metal ion toxicity in *Mycobacterium tuberculosis*, in influencing the extrusion of multiple structurally unrelated drugs and enhancing the biofilm formation of *Escherichia coli* and *Mycobacterium smegmatis.* Overexpression of Rv3270 increased the tolerance of host cells to norfloxacin, ofloxacin, sparfloxacin, ampicillin, oxacillin, amikacin and isoniazid. A significantly lower accumulation of norfloxacin, ethidium bromide, bocillin FL and levofloxacin in cells harbouring Rv3270 as compared to host cells indicated its role in enhancing efflux activity. Although over-expression of Rv3270 did not alter the susceptibility levels of levofloxacin, rifampicin and apramycin, the presence of a sub-inhibitory concentration of Zn^2+^ resulted in low-level tolerance towards these drugs. Of note, the expression of Rv3270 enhanced the biofilm-forming ability of the host cells strengthening its role in antimicrobial resistance. Therefore, the study indicated that the over-expression of Rv3270 enhances the drug efflux activity of the micro-organism where zinc might facilitate drug–metal cross-resistance for some antibiotics.

## **Graphical abstract**: The probable model depicting antibiotic export by Rv3270

(a) Antibiotics are reported to enter the cells through porin channels such as Msp family porins .(b) Zn^2+^ (green dots) imported into the cells through probable Zn^2+^ importers such as ZIP transporters. (c) Zinc inside the cells may influence the efflux of antibiotics by a co-transportation process through Rv3270 triggered by ATP hydrolysis. (d) Rv3270 possibly captures beta-lactams and extrudes them out of the cells via hypothetical linking proteins (outer membrane efflux adaptor proteins) and porins (Msp family) like AcrAB-TolC and Rv0194. This maintains a lower concentration of the antibiotic inside the cell, reducing its chances of exerting inhibitory effects on cell wall synthesis.
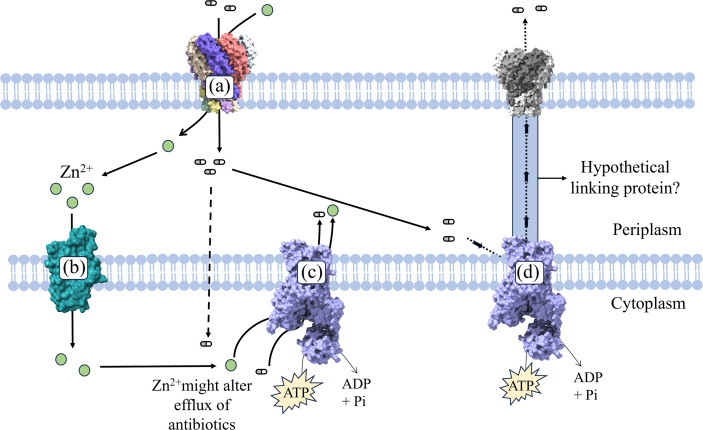

## Introduction

Globally, multi-drug-resistant *Mycobacterium tuberculosis* represents a leading cause of mortality. There are multiple challenges of combating such disease. Along with cell wall impermeability, alterations of drug targets, mutations affecting enzymes, various active efflux systems expelling antibiotics and toxins are also major contributors to intrinsic drug resistance [1, 2]. Apart from extruding antibiotics, toxins, quorum-sensing molecules and adhesins, efflux pumps such as ctpV of *M. tuberculosis*, CzcA protein from *Ralstonia metallidurans* and CopA from *Escherichia coli* also regulate the cellular concentration of metal ions involved in various fundamental metabolic, physiological and biochemical pathways for the survival and virulence of the bacterium [3–7]. There are multiple reports describing the dual-activity efflux pumps as a cause of imparting resistance to antibiotics and eliminating metal toxicity. One such finding revealed the role of an established NiCoT family metal transporter, NicT (rv2856) of *M. tuberculosis*, in influencing antibiotic resistance [8]. It is interesting to note that the Czcs–CzcR two-component system in controlling expression of the CzcCBA efflux pump system is reportedly involved in heavy metal resistance such as with zinc, cadmium and cobalt along with imparting resistance towards carbapenems such as imipenem [9]. Similarly, the *tcrB* gene of *Enterococcus faecium*, reported to have originated from *E. coli* of the CPx type ATPase metal transporter family, confers resistance to copper along with macrolide and glycopeptide resistance [10]. Moreover, MacAB efflux pump protein of *Agrobacterium tumefaciens* is found to confer resistance to arsenite along with penicillin, a macrolide [11].

P-type ATPases comprise a class of active membrane proteins that are auto phosphorylated at the conserved aspartate residue during its catalytic activity of regulating the concentration of various metal ions against their electrochemical gradients by utilizing ATP hydrolysis [12]. Genome analysis of *M. tuberculosis* has revealed that these transporters are conserved and present in abundance, and 12 P-type ATPase transporters have so far been detected [13]. P-type ATPase consists of multiple domains such as the three cytoplasmic domains, namely the actuator, nucleotide binding, phosphorylation domains and the transport domains that are embedded in the membrane [14]. These transporters are known to create a balance and regulate metal concentrations within the cells to avoid excessive accumulation of metals that might lead to toxicity. The copiousness of such proteins implies that these might play crucial roles in the survival and virulence of the micro-organisms. A previous report suggested that these types of transporters might also play an important role in drug–metal cross-resistance such as P-type ATPase and MdrL efflux pump-mediated drug resistance that is observed in estuarine isolates of *Pseudomonas* sp. and *Vibrio* sp*.* [15]. Previous reports established that two P-type ATPases, Rv3270 and Rv1469, were essential for murine infection by *M. tuberculosis* [16]. According to other reports based on micro-array analysis, Rv3270 and Rv1997 are among the metal transporters that are induced and over-expressed due to treatment with anti-tubercular drugs such as isoniazid, under the stress conditions of SDS, sodium hypochlorite and hypoxic starvation conditions [14, 17, 18]. It was particularly noted that Rv3270 played a role during *M. tuberculosis* infection of murine models that its expression levels were induced during stress conditions. Given the above, in the present study with the help of molecular and *in vivo* expression analyses such as of antibiotic sensitivity assessments, intracellular dye and antibiotic accumulation, semi-quantitative biofilm formation and growth curve, and *in silico* analyses (e.g. molecular docking) we attempted to establish the role of a putative metal transporter of *M. tuberculosis*, Rv3270, in influencing multiple drug efflux activities of *Mycobacterium smegmatis* and *E. coli*, which might be extrapolated to understand its performance in *M. tuberculosis*.

## Methods

### Bacterial strains, plasmids and media

The bacterial strains used in this study were *E. coli* XL1-Blue, *E. coli* CS109 and *M. smegmatis* MC^2^ 155 (ATCC) [19]. The *E. coli* XL1-Blue strains were cultured in Luria–Bertani broth (LB) and agar (Hi-Media) in the presence of 12.5 µg ml^−1^ tetracycline. *E. coli* strains harbouring pBAD18-Cam were grown in the presence of 20 µg ml^−1^ chloramphenicol [20]. *M. smegmatis* MC^2^ 155 was grown in Middle Brook 7H9 broth and 7H11 agar medium (Sigma-Aldrich) supplemented with oleic acid-ADC enrichment (Hi-Media), 0.35 % (w/v) glycerol, 0.05 % (w/v) Tween 80 and antibiotics (50 µg ml^−1^ kanamycin and 100 µg ml^−1^ hygromycin for the strains harbouring pMIND vector) [21]. Cation-adjusted Mueller–Hinton broth (MH) was used for antibiotic susceptibility testing of *E. coli* strains (Hi-Media). All the restriction endonucleases were purchased from New England Biolabs. Unless otherwise specified all the other reagents, including antibiotics, were purchased from Sigma-Aldrich.

### Construction of recombinant plasmids for *in vivo* studies

Rv3270 was amplified by PCR from the genomic DNA of *M. tuberculosis* H37Rv using specific primers. The primers were as follows: for cloning in pBAD-18Cam vector – FP 5′-CTCTCTGCTAGCAGGAGGCTCTCTCTATGACCCTGGAAGTG-3′, RP 5′-CTCTCTAAGCTTCTAAGCGGTCCAGGCGGTAGCGG-3′ and for pMIND vector – 5′-CTCTCTCATATGAGGAGGCTCTCTCTATGACCCTGGAAGTG-3′, RP 5′-CTCTCTACTAGTCTAAGCGGTCCAGGCGGTAGCGG-3′. The amplicon was cloned within sites *Nhe*I*–Hin*dIII in the pBAD-18Cam (*Cam* ^R^) vector [20] and within *Nde*I*–Spe*I in the pMIND vector to create pD3270 and pM3270, respectively [21]. *E. coli* XL1-Blue and CS109 cells were transformed with the construct pD3270 whereas *M. smegmatis* cells were transformed with pM3270. The plasmid, pD3270, was expressed in CS109 by inducing the cells with 0.2 % (w/v) arabinose. On the other hand, expression of pM3270 was obtained by inducing the cells with 20 ng ml^−1^ tetracycline in *M. smegmatis* [22].

### Expression of putative transporter Rv3270 in *E. coli*

Using an overnight culture (0.1 %), 10 ml LB broth was inoculated and incubated at 37 °C with shaking at 150 r.p.m until the culture reached an OD_600_ of 0.2. Expression of the gene and subsequent protein production was induced with 0.2 % arabinose and incubated for 14–16 h. The cells were spun down at 10 000 ***g*** for 10 min at 4 °C in Eppendorf 5810R. The supernatant was discarded and the pellet was washed with 1 ml 10 mM Tris/Cl buffer (pH 7.5) and resuspended in the same buffer. A protease inhibitor, PMSF, was added at a final concentration of 1 mM to the cell suspension and it was sonicated with five pulses of 45 s each in a Corning CoolRack M6 placed in ice, followed by centrifugation at 16 000 ***g*** for 2 min. The supernatant fraction was collected and further centrifuged at 4 °C for 1 h at 16 000 ***g***. The supernatant was conserved and the pellet fraction was further resuspened in 100 µl of Tris/Cl buffer (pH 7.5). The pellet fraction was treated with 2 % (w/v) sarcosyl (sodium lauroyl sarcosinate), mixed thoroughly and incubated at 37 °C on a thermomixer (Eppendorf) with shaking for 1 h to solubilize the membrane proteins. The sample was further centrifuged at 16 000 ***g*** for 1 h at 4 °C and the supernatant was recovered. The proteins in recovered supernatant were analysed through SDS-PAGE (12 % acrylamide) [23].

### Antimicrobial susceptibility assay of various classes of drugs and metal salt

MICs of various structurally unrelated classes of antibiotics, such as fluoroquinolones, beta-lactams, tetracycline, anti-tubercular drugs, aminoglycosides, metal salts of magnesium, cobalt, and zinc, and natural dyes [such as ethidium bromide (EtBr), acriflavine and rhodamine B] were determined. The micro-broth dilution assay was performed in 96-well micro-titre plates [8]. The antibiotics, metal salts and dyes were two-fold serially diluted, and the total volume was made up to 300 µl with MH broth containing 0.2 % arabinose for *E. coli* whereas for *M. smegmatis* cells (10^5^ per well), the volume was made up to 200 µl with 7H9 media and 20 ng µl^–1^ tetracycline was used for induction of the gene. The optical density of the culture medium was measured after 12–16 h for *E. coli* and 48–72 h of incubation for *M. smegmatis*. To study the effect of zinc on antimicrobial susceptibility, ZnSO_4_ at a concentration of one-eighth the MIC was added to the media. Following CLSI guidelines [24], the optical density of the cultures was determined at 600 nm by using a Multiskan Spectrum spectrophotometer (model 1500; Thermo Scientific). The experiments were replicated six times for consistency.

### Ethidium bromide accumulation assay

This assay was carried out as mentioned earlier with minor modifications [25]. *M. smegmatis* cells (10^5^ cells), harbouring Rv3270 were washed with 50 mM phosphate buffer supplemented with 0.05 % Tween 80 and MgCl_2_. The cells were resuspended in the same buffer, OD_600_ was adjusted to 0.6 and, finally, energized with 25 mM glucose. EtBr was added to a final concentration of 1.5 µM and the accumulation of the dye was measured as relative fluorescence with an excitation wavelength of 530 nm and emission wavelength of 560–640 nm. After incubation of cells for 15 min in the presence of EtBr, reserpine was added at a concentration of 32 µg ml^−1^ to determine the blocking effect of the *Rauwolfia* alkaloid [26].

### Assessment of intracellular drug accumulation activity

The assays for norfloxacin, levofloxacin and bocillin FL accumulation were performed based on the protocol as described previously with some modifications [27, 28]. Cells expressing *rv3270* were grown up to an OD_600_ of ~0.6 and were washed three times with 50 mM phosphate buffer. *E. coli* cells were energized for 20 min at 30 °C with 0.2 % glucose. Fluoroquinolones (norfloxacin, levofloxacin) and bocillin FL were added to the reaction mixture at a final concentration of 10 mg l^−1^ and 10 µM, respectively. ZnSO_4_ was added to the culture medium at a concentration of one-eighth of its MIC value for the assessment of levofloxacin accumulation. After the requisite time, the aliquots were withdrawn (0.5 ml) and washed three times with phosphate buffer. The samples were resuspended in 0.1 M glycine hydrochloride buffer (pH 3.0) and incubated at 37 °C for 2 h for *E. coli* and overnight for *M. smegmatis* cells. The cells were further centrifuged and the fluorescence of the supernatant was measured by using a spectrofluorimeter (FluoroMax 4; HORIBA Scientific Instruments) for norfloxacin (at an excitation wavelength 281 nm and emission wavelength 447 nm), levofloxacin (excitation wavelength of 285 nm and emission wavelength of 496 nm) and for bocillin FL (excitation and emission wavelength of 488 and 530 nm, respectively). Relative efflux (RE) was calculated concerning the control strain as follows: RE=1+(N_control_ – N_test_)/N_control_, where N_control_ represents antibiotic accumulated by the control strain harbouring only the vector control and N_test_ represents antibiotic uptake by the cells harbouring Rv3270 [8]. The relative percentage of efflux was calculated by: (N_control_ − N_test_)/ N_control_ × 100.

### Growth curve analysis

Overnight cultures of *M. smegmatis* cells harbouring cloned Rv3270 and empty pMIND were harvested and washed twice with Middle Brook 7H9 medium supplemented with 0.05 % Tween 80 and 100 µg ml^−1^ hygromycin and finally diluted to an OD_600_ of ~0.04 with the same medium. Growth in the presence of a sub-inhibitory concentration of EtBr (1.5 µM) when induced with 20 ng µl^–1^ of tetracycline was determined by measuring the optical density at 600 nm using a spectrophotometer [Multiskan Spectrum spectrophotometer (model 1500; Thermo Scientific)] [29].

### Semi-quantitative assessment of biofilm formation

For biofilm formation, cells were grown in 24-well polystyrene plates. To determine their effect on biofilm formation, the experiment was conducted both in the presence and absence of metal salts. Briefly, *M. smegmatis* and *E. coli* cells were grown overnight in M63 medium and LB medium, respectively [30, 31]. The next day, the cultures were washed with PBS and resuspended in the aforementioned media with an OD_600_ of ~0.6. Each well of the plates containing 1× M63 medium (for *M. smegmatis*) and one-fifth LB (for *E. coli*) were inoculated with the absorbance optimized cell suspensions, and incubated at 37 °C for 48 h. The planktonic cells were washed with PBS and the plates were kept for drying. Crystal violet (CV) at a concentration of 0.1 % was added to each well and kept for 15 min at room temperature. The stain was removed and the wells were washed with water followed by destaining with 1 ml of 33 % glacial acetic acid. The absorbance of the CV in the destained solutions was measured using a Multiskan Spectrum spectrophotometer (model 1500; Thermo Scientific) at 600 nm. The biofilm formation index was calculated by: BFI=(OD_600_ of acetic acid solubilized CV of culture – OD_600_ of acetic acid solubilized CV of control)/OD_600_ of planktonic cells. To ascertain the effect of the inhibitor (reserpine) and zinc on biofilm formation in Rv3270 harbouring *M. smegmatis*, the experiment was repeated with reserpine in 24-well plates as described previously [32, 33]. Each well of the plate was filled with 1 ml of M63 medium, supplemented with 32 µg ml^−1^ of reserpine and one-eighth MIC of Zn^2+^, and inoculated with *M. smegmatis* cells. The plates were incubated for 3 days at 37 °C. After incubation, the wells were washed with sterile PBS and stained with 0.1 % CV dye for 15 min. Thereafter, the wells were again washed and destained with 33 % glacial acetic acid and, finally, we measured the absorbance at OD_600_ using a Multiskan Spectrum spectrophotometer (model 1500; Thermo Scientific). BFI was calculated to determine the effect of Zn^2+^ by: BFI=(OD_600_ of acetic acid solubilized CV of culture – OD_600_ of acetic acid solubilized CV of control)/OD_600_ of planktonic cells supplemented with zinc. The percentage of biofilm inhibition was calculated as: [A_control_ – A_treated_)/A_control_]×100 [34]. All the biofilm experiments were replicated six times for consistency.

### *In silico* analysis of Rv3270

The three-dimensional structure of Rv3270 was retrieved from the AlphaFold database [35]. Subsequently, it was energy minimized using the amber14ff force field with 500 steps of conjugate gradient and 100 steps of gradient descent. For molecular docking, Autodock Vina-1 was used [36] whereas UCSF ChimeraX was utilized for structure analysis and visualization [37]. The domain annotation of the tertiary structure was completed by using the InterPro server. Furthermore, Schrodinger Maestro was used to generate images of the protein–ligand interactions [38].

### Statistical analysis

All experiments relating to the accumulation of antibiotics and dyes were performed in triplicate and the results were calculated as mean±sd. The statistical significance (*P* value) was calculated using GraphPad by performing two-sample, unpaired Student’s t-tests where *P<0.01, **P<0.001 and ***P<0.0001.

## Results

### Over-expression of Rv3270 decreased the susceptibility toward various classes of antibiotics

**Fig. 1.**
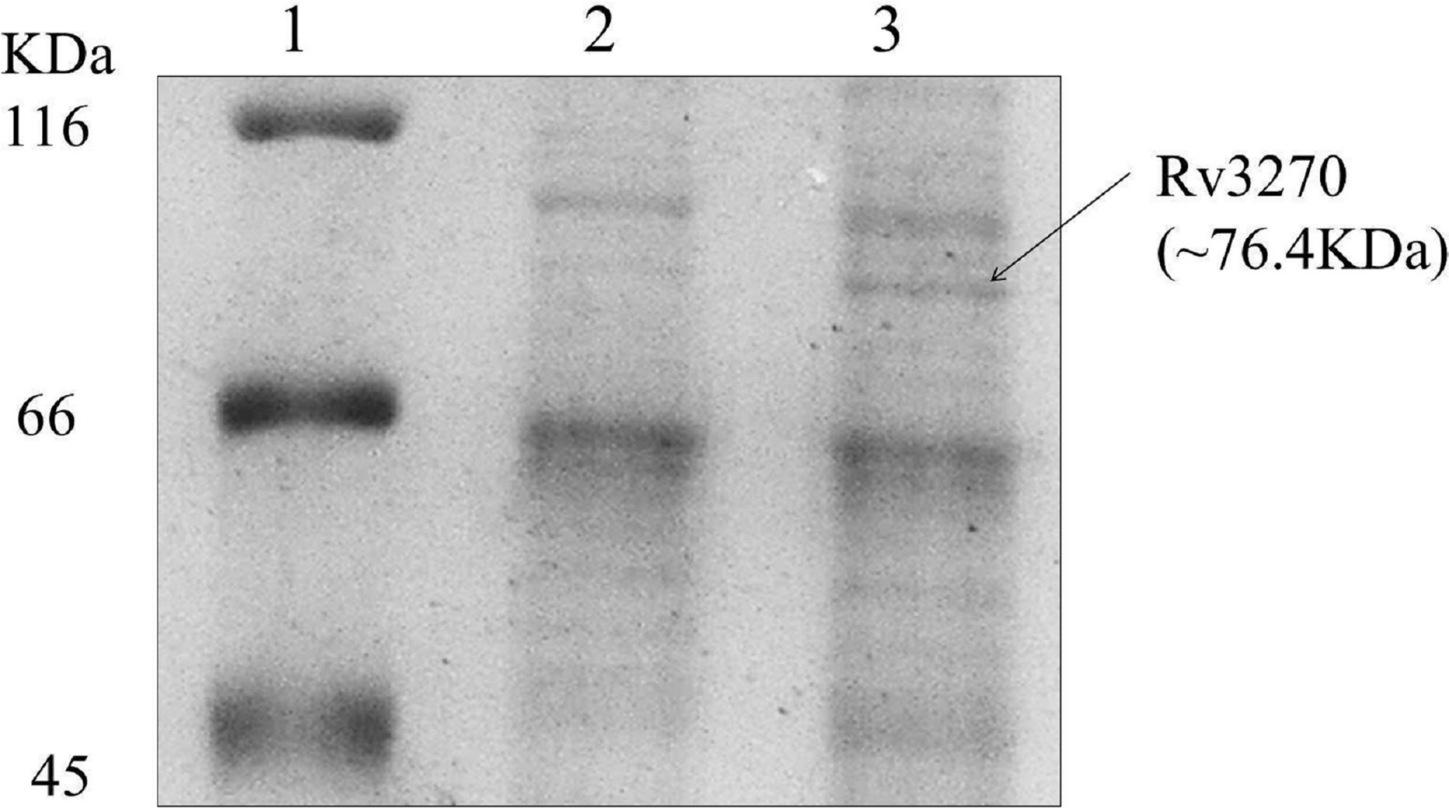
SDS-PAGE image of solubilized membrane fraction of *E. coli* CS1O9 cells harbouring pD3270 upon induction with 0.2 % arabinose. Lane 1: unstained protein molecular weight marker (Thermo Scientific), Lane 2: uninduced pD3270 in CS1O9 cells, Lane 3: *E. coli* CS1O9 cells expressing *rv3270*.

To establish the activity of Rv3270 in multidrug resistance, the change in susceptibility towards antibiotics upon its ectopic expression was determined. The gene *rv3270* was expressed in *E. coli* CS109 (Fig. 1) and *M. smegmatis* MC^2^155 (Fig. S1, available in the online version of this article), and their antibiotic susceptibilities towards various structurally unrelated antibiotics and compounds were assayed. The susceptibilities were changed many-fold towards various classes of structurally unrelated antibiotics, metal salts and dyes upon expression of Rv3270. Ectopic expression of *rv3270* increased resistance towards fluoroquinolones, namely norfloxacin (fourfold), ofloxacin (fourfold) and sparfloxacin (fourfold) with respect to the control. Decreased susceptibilities were observed for beta-lactams, namely ampicillin (fourfold), oxacillin (fourfold) and amikacin (twofold), for *E. coli* cells (Table 1), which could be correlated with the observed values for *M. smegmatis* cells. However, *M. smegmatis* cells showed additional susceptibilities towards isoniazid (eightfold) (Table 2). According to he literature, Rv3270 acts a zinc transporter [39, 40]. Here, in the presence of zinc (ZnSO_4_), we observed a fold change in susceptibility towards those antibiotics that did not show any change in resistance in the absence of zinc. A range of zinc concentrations (3200–3.125 µM) along with antibiotic concentrations ranging from 8 to 0.0625 µg ml^–1^ was tested, and in the presence of a sub-inhibitory concentration of zinc (30 ppm), resistance of levofloxacin was increased by fourfold, and for apramycin it was increased by twofold in *E. coli* cells expressing Rv3270 (Table 1). For *M. smegmatis* cells expressing *rv3270*, the MICs were co-related to those observed for *E. coli* cells. For rifampicin, *M. smegmatis* cells showed a twofold decrease in susceptibility (Table 2). However, the presence of zinc had no additional effect on norfloxacin, sparfloxacin, ampicillin, oxacillin, amikacin and isoniazid (Table S1).

**Table 1.** Minimum inhibitory concentrations of various unrelated classes of antibiotics against CS109 cells carrying pBAD18 Cam vector (control of *E. coli* cells), and CS109 cells harbouring Rv3270 (pD3270) in the presence and absence of inhibitor reserpine (32 µg ml^−1^)

Antibiotic	MIC (mg l^−1^)			
			Reserpine (32 µg ml^−1^)	
	*E. coli* CS109	CS1O9/pD3270	*E. coli* CS1O9	CS1O9/pD3270
Norfloxacin	0.062	0.25	0.015	0.015
Ofloxacin	0.062	0.25	0.031	0.062
Sparfloxacin	0.012	0.05	0.003	0.006
Ampicillin	16	64	4	4
Oxacillin	8	32	4	8
Amikacin	4	8	2	2
Levofloxacin	0.05	0.05	0.05	0.05
Levofloxacin+Zn^2+^	0.05	0.2	0.025	0.025
Apramycin	8	8	8	8
Apramycin+Zn^2+^	8	16	8	16

**Table 2.** Minimum inhibitory concentrations of various unrelated classes of antibiotics against *M. smegmatis* cells harbouring pMIND vector (control of *M. smegmatis* cells) and Rv3270 (pM3270) in the presence and absence of reserpine (32 µg ml^−1^)

Antibiotic	MIC (mg l^−1^)			
			Reserpine (32 µg ml^−1^)	
	*M. smegmatis* MC^2^155	*M. smegmatis* MC^2^155/pM3270	*M. smegmatis* MC^2^155	*M. smegmatis* MC^2^155/pM3270
Norfloxacin	2	8	0.5	0.5
Ofloxacin	0.5	2	0.5	1
Sparfloxacin	2	8	1	2
Ampicillin	32	128	8	8
Oxacillin	16	64	8	16
Amikacin	4	8	2	2
Isoniazid	32	256	8	8
Levofloxacin	2	2	2	2
Levofloxacin+Zn^2+^	2	8	1	1
Apramycin	8	8	8	8
Apramycin+Zn^2+^	8	16	8	16
Rifampicin	1	1	1	1
Rifampicin+Zn^2+^	1	2	0.5	0.5

To understand whether it was the zinc rather than the sulphate ion that was responsible for the variations in susceptibility, the entire set of experiments was repeated in the presence of magnesium sulphate (MgSO_4_) and cobalt chloride (CoCl_2_). There was no change in MICs, thus negating the possibility of the involvement of sulphate ions in the experiment. On the other hand, addition of reserpine increased the susceptibility towards antibiotics. In comparison to the vector control, reserpine had an inhibitory effect on *E. coli* and *M. smegmatis* cells expressing *rv3270*. After addition of reserpine the MICs of the antibiotics (e.g. norfloxacin, ampicillin, amikacin, levofloxacin, isoniazid and rifampicin) were equal for cells expressing *rv3270* and the vector control indicating an inhibitory effect on Rv3270. Cells expressing *rv3270* were comparatively less susceptible to ofloxacin, sparfloxacin and oxacillin, though the fold change was reduced to twofold as compared to two- to eightfold differences as observed in the absence of reserpine (Table 1). However, there was no change in susceptibility for apramycin, which could possibly be due to low dosage of the inhibitor (Table 2). Therefore, the decrease in susceptibility for the various antibiotics in cells expressing *rv3270* indicates the involvement of Rv3270 in conferring multidrug resistance.

### Intracellular concentration of antibiotics was reduced upon ectopic expression of *rv3270*

**Fig. 2.**
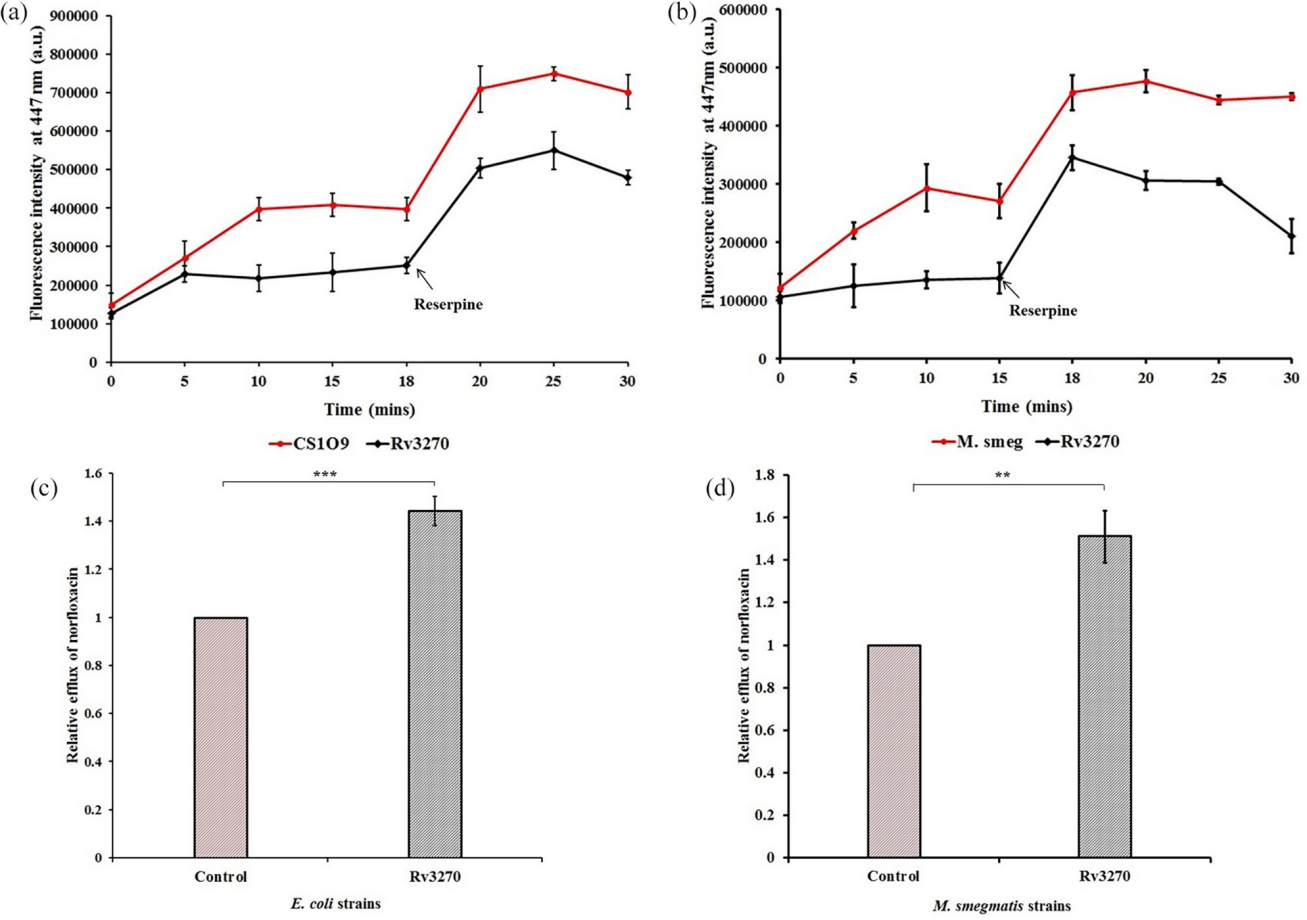
Intracellular accumulation of norfloxacin in *E. coli* (**a**) and in *M. smegmatis* (**b**) cells harbouring Rv3270 in comparison to control cells (harbouring the empty vector) with respect to time. Norfloxacin was added at 0 min. Sample aliquots were drawn every 5 min for a period of 30 min and reserpine was added at 18 min. Relative efflux was calculated after exposing the *E. coli* (**c**) and *M. smegmatis* (**d**) cells to norfloxacin for 10 min. A two-tailed unpaired Student’s t-test was performed with the control and test datasets: **P<0.001, ***P<0.0001. The *P* values were 0.0001 (a) and 0.0018 (**b**) respectively. Error bars indicate mean±sd.

**Fig. 3.**
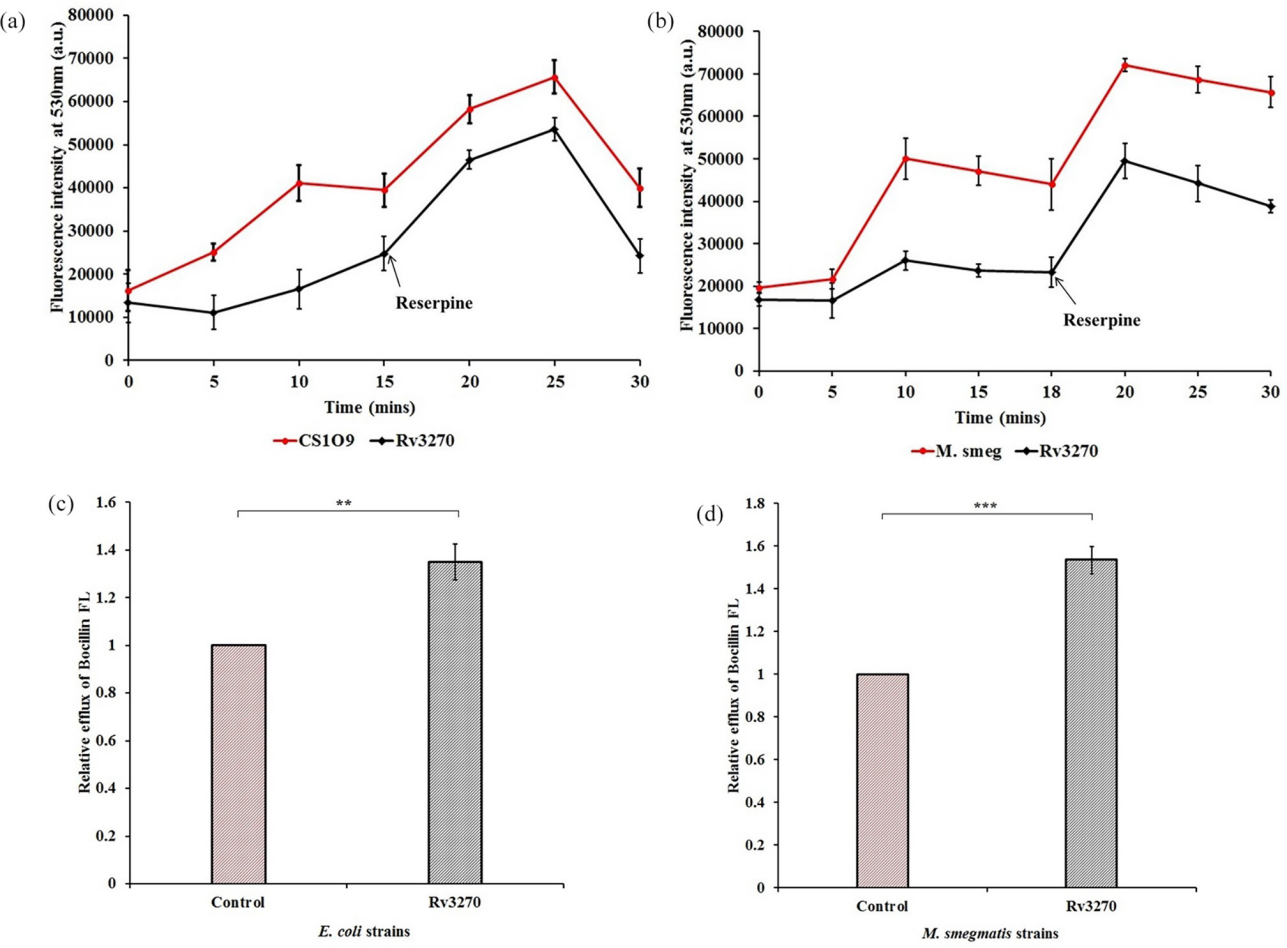
Intracellular accumulation of bocillin FL in *E. coli* (**a**) and in *M. smegmatis* (**b**) cells harbouring Rv3270 in comparison to control cells (harbouring the empty vector) with respect to time. Bocillin FL was added at 0 min and sample aliquots were drawn every 5 min for a period of 30 min and reserpine was added at 15 and 18 min for *E. coli* and *M. smegmatis* cells respectively. Relative efflux was calculated after exposing the *E. coli* (**c**) and *M. smegmatis* (**d**) cells to bocillin FL for 15 min. A two-tailed unpaired Student’s t-test was performed with the control and test datasets: ** *P*<0.001, ***P<0.0001. The *P* values were 0.001 (a) and 0.0001 (b) respectively. Error bars indicate mean±sd.

To further confirm that the increase in resistance towards antibiotics was due to the efflux activity of Rv3270, an intracellular drug accumulation assay was performed. Accumulation of norfloxacin (a representative of the fluoroquinolone group) was lower for the *E. coli* strain harbouring Rv3270 (when induced with 0.2 % arabinose) as compared to the control strain (Fig. 2a). A similar outcome was observed for the tetracycline-induced *rv3270* clone expressed in *M. smegmatis* cells (Fig. 2b). Accumulation was decreased by 44–50 % after 10 min of antibiotic exposure for both strains (Fig. 2c, d). The addition of reserpine led to a significant increase in fluorescence signal in both the experimental as well as in the control strains, and the relative efflux percentage of cells harbouring Rv3270 decreased to 20–25 % after 5 min of reserpine exposure to the cells. Likewise, bocillin FL was used as a substrate to determine the accumulation activity of beta-lactams. Accumulation of bocillin FL was notably lowered after 15 min of exposure to *E. coli* and *M. smegmatis* cells carrying Rv3270 (Fig. 3a, b). The relative decreases in accumulation levels in *E. coli* and *M. smegmatis* were 35 and 53 %, respectively (Fig. 3c, d). The addition of reserpine decreased the relative efflux percentage of bocillin FL by *E. coli* to 20 % and *M. smegmatis* to 30 % at 15 and 18 min respectively.

**Fig. 4.**
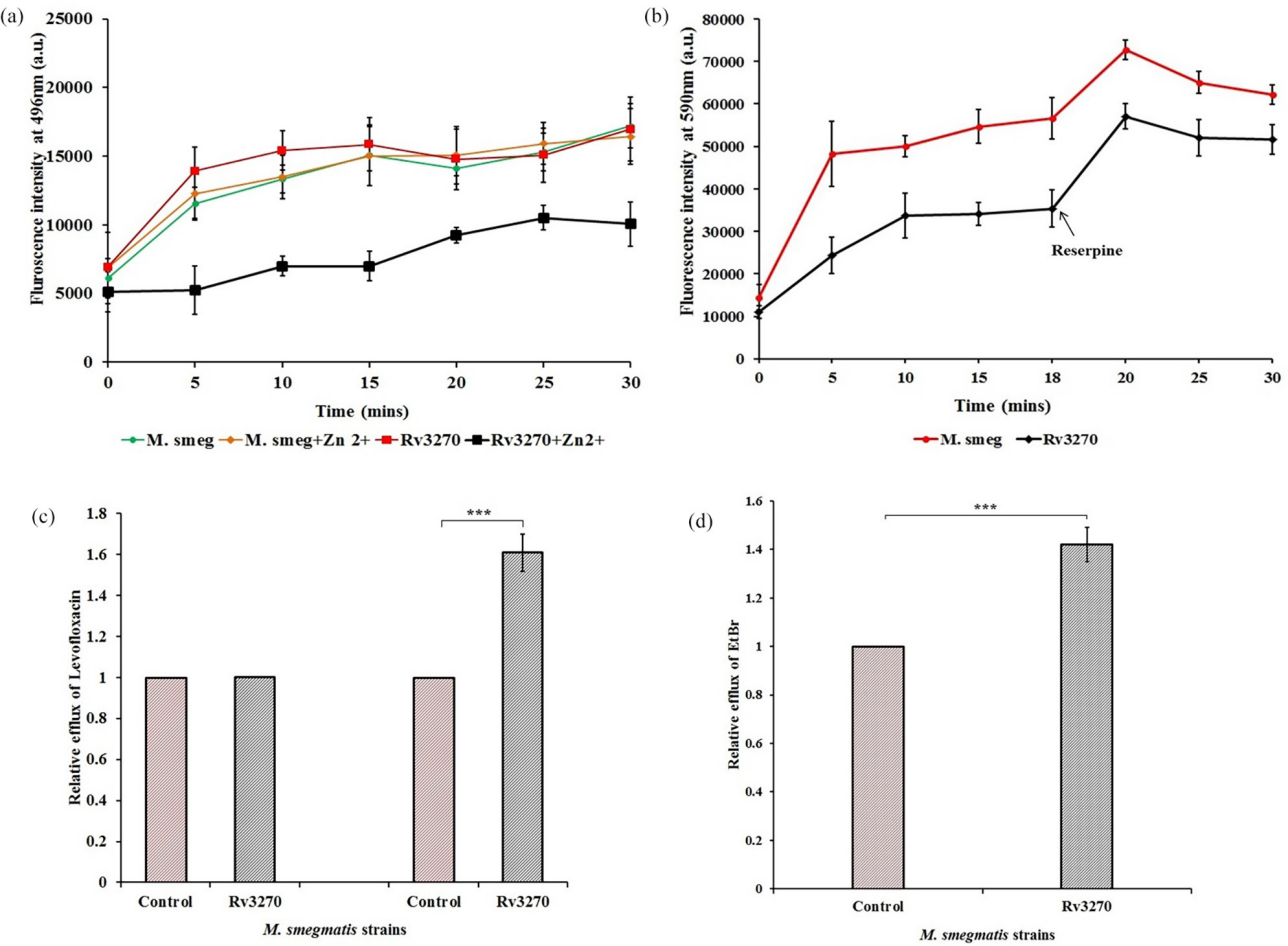
Intracellular accumulation of levofloxacin and EtBr in *M. smegmatis* cells harbouring Rv3270 in comparison to control cells (harbouring the empty vector) with respect to time (**a and b**). Levofloxacin accumulation in *M. smegmatis* cells in the presence and absence of Zn^2+^ was determined (**a**). The substrates were added at 0 min and sample aliquots were drawn every 5 min for a period of 30 min. To determine the effect of inhibitor on efflux activity of Rv3270, EtBr (substrate) was added at 0 min and reserpine, an inhibitor, was added at 18 min (**b**) and sample aliquots were collected every 5 min followed by fluorescence determination at 590 nm. Relative efflux was calculated after exposing the *M. smegmatis* cells to levofloxacin and EtBr for 15 min (**c and d**). A two-tailed unpaired Student’s t-test was performed with the control and test datasets: *** *P*<0.0001. The *P* values were 0.0003 (a) and 0.0001 (b) respectively. Error bars indicate mean±sd.

Since it was previously noted that Rv3270 facilitates maintenance of zinc homeostasis [39], and there was no change in MIC value as well as the level of accumulation of levofloxacin in the absence of zinc with respect to the control cells, the influence of zinc on the accumulation of levofloxacin was determined. In the presence of zinc, a fourfold increase in resistance toward levofloxacin was observed. Accordingly, the effect of zinc on levofloxacin accumulation was also determined. A sub-inhibitory concentration (30 ppm) of zinc led to a decrease of 55 % in the accumulation of levofloxacin after 15 min of drug–cell contact, indicating the role of zinc in acquiring resistance by influencing the efflux activity of Rv3270 (Fig. 4a, c). There was a consistent and significant decrease in the accumulation of antibiotics in the cells harbouring Rv3270 as observed from the relative efflux data, indicating a role of Rv3270 in influencing multi-drug efflux activity.

### Expression of *rv3270* reduces EtBr accumulation in mycobacteria

To investigate the role of Rv3270 in enhancing the efflux of toxic substances such as EtBr, an accumulation assay was performed. Usually, non-toxic concentrations of EtBr have been widely used as a substrate to evaluate the efflux activity of a protein [41]. It fluoresces strongly inside the cellular components while undergoing attenuation in contact with an aqueous environment outside the cell [25]. Therefore, a reduction in the fluorescence within the cells indirectly indicates an efflux activity of EtBr (Fig. 4b). After 15 min of exposure of the substrate to the cells, the accumulation level of EtBr was reduced by 40 % as compared to the control (Fig. 4d). To further verify whether the lower levels of accumulation were due to the effect of the expression of Rv3270, an efflux pump inhibitor, reserpine, was used [26]. The addition of reserpine led to a significant increase in fluorescence signal in both the experimental as well as in control strains, and the relative efflux percentage decreased to 20 % after 20 min. Since a sub-inhibitory concentration of reserpine was added, and the difference in levels of accumulation between the test and control set persisted, we speculate a probable influence of Rv3270 on the efflux of EtBr, which could be extrapolated to the elimination of toxic substances from the cells as well.

Next, to check whether EtBr had any effect on the growth of *M. smegmatis* cells, the same was determined and it was noted that in the absence of EtBr, there was no change in growth for the experimental and control cells. However, in the presence of 1.5 µM EtBr, there was a notable decrease in the growth rate of wild-type *M. smegmatis* as compared to the strain overexpressing Rv3270. Furthermore, growth of both strains was completely diminished in the presence of 256 µg ml^−1^ reserpine (Fig. S2). Overall, the above findings indicate the influence of Rv3270 in extruding the toxic compounds.

### Over-expressed Rv3270 enhances biofilm formation

**Fig. 5.**
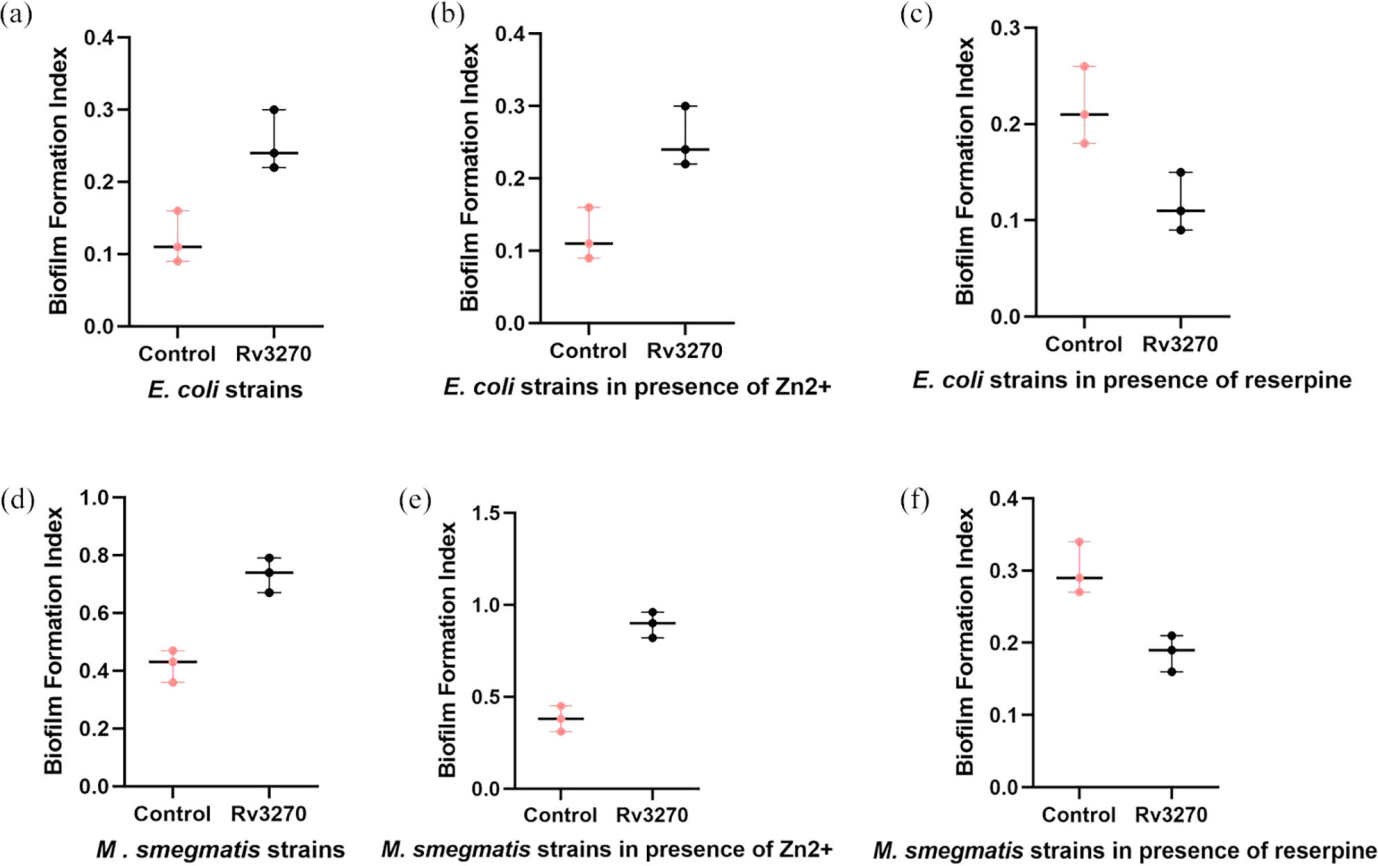
Semi-quantitative biofilm formation assay: biofilm formation by *E. coli* CS1O9 harbouring Rv3270 (**a**), *E. coli* CS1O9 cells expressing Rv3270 supplemented with Zn^2+^ (**b**) and Rv3270-overexpressing *E. coli* cells in the presence of reserpine (**c**) was calculated. Similarly BFI was calculated for *M. smegmatis* over-expressing Rv3270 (**d**), and in the presence of Zn^2+^ (**e**) and reserpine (**f**) with respect to the vector control by the CV staining method. A two-tailed unpaired Student’s t-test was performed with the control and test datasets: *P<0.01, **P<0.001, ***P<0.0001.

Efflux pumps often contribute to antibiotic resistance by extruding toxins out of the cells and enhancing the aggregation of cells by regulating EPS molecules, adhesins and quorum-sensing molecules [42, 43]. To understand the effect of Rv3270 on the biofilm formation of *E. coli* and *M. smegmatis*, a static biofilm quantification assay was performed in the presence and absence of an inhibitor. BFI values for the control and *rv3270*-expressing cells were 0.35 and 0.51 for *E. coli*, and 0.42 and 0.73 for *M. smegmatis*, respectively (Fig. 5a, d). A two- to fourfold increase in biofilm formation was observed for cells expressing *rv3270*. In the presence of Zn^2+^ a similar fourfold increase of BFIs was observed for both *E. coli* and *M. smegmatis* (Fig. 5b, e). It was previously reported that reserpine has an inhibitory effect on the biofilm formation of *Staphylococcus aureus* [34] and a similar finding was observed here. In the presence of 32 µg ml^−1^ reserpine, the biofilm-forming ability of the cells was reduced. The reduction of biofilm formation for *rv3270* over-expressing cells was greater as compared to the host cells. BFI values for the control and experimental cells were 0.2 and 0.181 for *E. coli*, and 0.3 and 0.18 for *M. smegmatis* (Fig. 5c, f). Taken together, the overall decrease in biofilm formation was 40 % for *E. coli* and 47 % for *M. smegmatis* cells. It was of note that the effect of *rv3270* was analogous to that in the Gram-negative *E. coli* system. The decrease in BFI of cells expressing *rv3270* was greater as compared to the control in the presence of reserpine, further validating the impact of *rv3270* on the host. This implies that Rv3270 might play a role in influencing the biofilm-forming ability of the micro-organism.

### Molecular docking analysis provides support for the multidrug efflux function of Rv3270

**Fig. 6.**
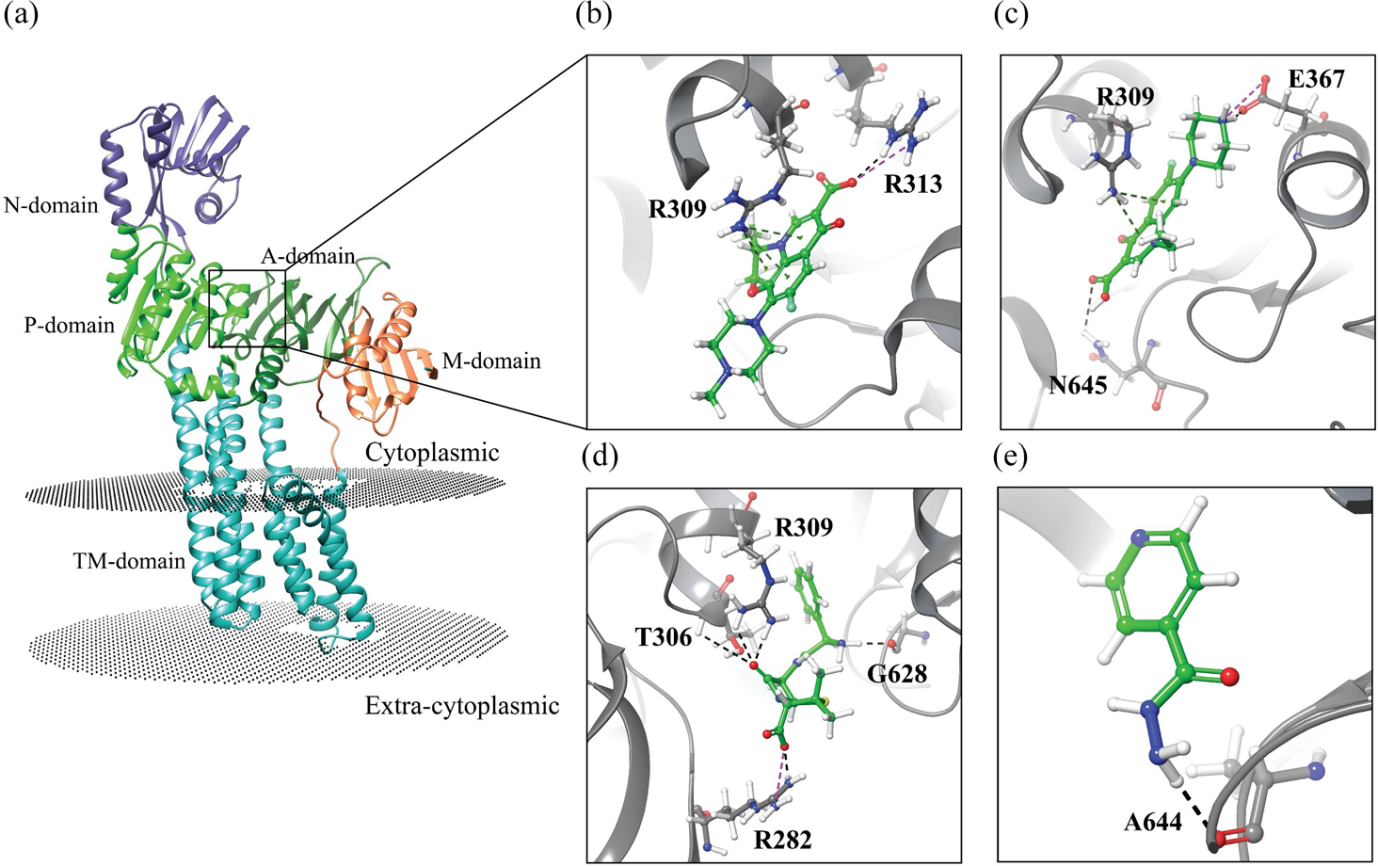
Fig. 4 Predicted tertiary structure and binding poses of Rv3270 (**a**). The AlphaFold predicted structure of Rv3270 reveals its distinct components, including the metal binding domain (M-domain) (coral), A-domain (dark green), P-domain (green), N-domain (dark slate blue) and eight *trans*-membrane helices (TM-domain). (**b–e**) Binding pose of levofloxacin, norfloxacin, ampicillin and isoniazid respectively. Black dotted line represents the H-bond, pink dotted line represents the salt bridge and green dotted line represents the cation–pi interaction. The possible membrane position was predicted using the PPM3.0 server.

Rv3270 is a cation-transporting P-type ATPase that transports divalent metals. Biochemically, Mn^2+^ transport was preferred over other divalent cations such as Zn^2+^ which is transported at a slower rate [44]. It was subsequently hypothesised that PacL1, a probable metallo-chaperone, increases the velocity of Zn^2+^ transport [39]. However, our *in vivo* analysis uggested that overexpression of *rv3270* leads to an increased efflux of specific antibiotics. To investigate the structure–function relationship, we conducted a study wherein we modelled the tertiary structure of Rv3270 and performed docking simulations with levofloxacin, norfloxacin, ampicillin and isoniazid. The tertiary structure of Rv3270 consists of four cytoplasmic domains, namely a metal-binding domain spanning residues 10–78, an actuator domain (A-domain) from 227 to 313, a nucleotide-binding domain (N-domain) from 417 to 540, and a phosphorylation domain (P-domain) encompassing residues 388–416 and 541–658 (Fig. 6a). Additionally, it contains a *trans*-membrane domain composed of eight membrane-spanning helices. Three cytoplasmic domains (except the metal-binding domain) and the *trans*-membrane domain are conserved in most of the P-type ATPase (Fig. S3) [45]. We conducted a blind docking study to investigate the interaction between antibiotics and Rv3270. The entire receptor was included in the grid box, and we generated ten poses for each antibiotic. Based on the binding free energy, we selected the best pose. Interestingly, the best binding poses for all the antibiotics were between the A-domain and P-domain. Among the antibiotics tested, levofloxacin exhibited the highest binding affinity (−7.2 kcal mol^–1^), followed by norfloxacin (−6.6 kcal mol^–1^), ampicillin (−6.3 kcal mol^–1^) and isoniazid (−4.2 kcal mol^–1^). Levofloxacin forms three cation–pi interactions with Arg309 and an H-bond, and a salt bridge with Arg313 (Fig. 6b). In the case of norfloxacin, binding was facilitated by Asn645 through H-bonding, Glu637 through H-bonding and salt bridge interaction, and Arg309 through two cation–pi interactions (Fig. 6c). Ampicillin interacted with Arg309, Thr306, Arg282 and Gly628 through H-bonding, with Arg282 also forming a salt bridge (Fig. 6d). Isoniazid, which exhibited the lowest affinity for Rv3270 among the docked antibiotics, formed a single H-bond with Ala644 (Fig. 6e). Furthermore, we utilized the MOLE2 server [6] in pore mode to predict possible channels in Rv3270 [46]. Interestingly, the binding site of the antibiotics is located at the entrance of the predicted pore on the cytoplasmic face (Fig. S4). While the detailed mechanism remains unexplored in our present work, the binding of antibiotics at the entrance of the predicted pore supports the hypothesis that Rv3270 could efflux antibiotics along with metal cations.

### Discussion

The role of the P-type ATPase Rv3270 as a metal transporter is well established in *M. tuberculosis* [39, 44]. In addition to the up-regulation of Rv3270 during metal toxicity, preferential activation of the gene in the presence of toxic compounds and the anti-tubercular drug isoniazid has also been observed [17]. Its probable role in anti-microbial and toxic compound efflux has not been widely explored. In the present work, we observed that in the presence of over-expressing *rv3270*, the susceptibility of isoniazid is decreased in *M. smegmatis* and, similarly, the susceptibilities of fluoroquinolones, beta-lactams and aminoglycosides are decreased in both *M. smegmatis* and *E. coli*. This suggests that Rv3270 has a wide range of substrate specificity varying from fluoroquinolones to anti-tubercular drugs. This finding is relevant as the above-mentioned classes of drugs are often used to treat tuberculosis.

Initially *E. coli* is used as a model organism to study the activity of Rv3270 as *E. coli* is fast growing, and is easy to culture and genetically manipulate, though the cell surface architecture varies widely between *E. coli* and *M. tuberculosis* [47]. *E. coli* is a Gram-negative bacteria whereas the highly lipid rich *M. tuberculosis* is acid-fast [1, 48]. Since Rv3270 is a membrane-bound protein, changes in the cell surface composition often lead to difficulties in expressing the protein. Accordingly, a closer model organism, *M. smegmatis,* is considered throughout the study. *M. smegmatis* is a non-pathogenic, fast growing micro-organism which has similar cell envelope architecture to that of *M. tuberculosis*. Over-expression of Rv3270 was therefore studied in *M. smegmatis*. It is interesting to note that the effect of this mycobacterial efflux pump is comparable to that observed in the case of Gram-negative species such as *E. coli*

Through antibiotic accumulation assays with fluoroquinolones and beta-lactams, the role of Rv3270 is observed to influence multi-drug efflux activity. To further verify its role in *E. coli* and *M. smegmatis* an inhibitor, reserpine, a plant-derived efflux pump blocker, was introduced. Reserpine increased the accumulation of antibiotics in both the host and the experimental strains and there was a steady difference in levels of accumulation of the antibiotics, indicating that Rv3270 influences the efflux of antibiotics in *E. coli* and *M. smegmatis* and reserpine plays a role as an inhibitor. Interestingly, Adhikary *et al*. [8] emphasized that a nickel/cobalt efflux pump, NicT, might use the electrochemical gradient created by Ni^2+^ uptake to transport drugs. Similarly, we have observed that in the presence of varying concentrations of Zn^2+^, Rv3270 could enhance the efflux of levofloxacin, which otherwise had no effect in the absence of the metal ion in the concerned host. In both *E. coli* and *M. smegmatis*, a two- to fourfold consistent decrease in susceptibility is observed for levofloxacin, apramycin and rifampicin in the presence of the metal ion, indicating a possibility of synergy in the facilitation of antibiotic efflux by Zn^2+^. The transport of positively charged divalent cations due to ATPase activity often establishes an electrochemical gradient along the membrane. This ATP hydrolysis facilitates the co-transport of zinc and antibiotics [49]. Rv3270 may use the ion gradient to influence the extrusion of antibiotics from the host. Our *in silico* studies identified the potential binding site of antibiotics in Rv3270. It revealed that all antibiotics bind to a region between the A-domain and P-domain of the protein, which coincides with the cytoplasmic entrance of the predicted channel in Rv3270. This finding provides additional support for the hypothesis that Rv3270 may facilitate the efflux of antibiotics. In a recent study, ta correlation between the efflux of a substrate and its binding affinity was proposed [50]. In comparative terms, it is plausible that a substrate with higher binding affinity is less likely to be exported than a substrate with relatively low affinity, as relatively strong binding could potentially hinder the efflux process. In our *in vitro* studies, isoniazid exhibited an eightfold change in MIC, while ampicillin and norfloxacin showed fourfold changes. These findings align with our docking studies, wherein isoniazid demonstrated a higher binding free energy, indicating lower affinity as compared to ampicillin and norfloxacin. Levofloxacin, which is not exported by Rv3270 in the absence of metal, displayed the highest affinity among the tested antibiotics. This strong binding of levofloxacin to Rv3270 is likely to impede its efflux. Rv3270 is predicted to be a class of Zn^2+^ transporter in *M. tuberculosis* which contributes to maintaining zinc homeostasis in the phagosome [51]. Therefore, it is speculated that the presence of very low concentrations of zinc might create an electrochemical gradient in *E. coli* and *M. smegmatis* that is likely to affect the stability of the Rv3270–levofloxacin complex and ultimately facilitate its efflux, though this hypothesis requires further supporting evidence.

We also observed that over-expression of *rv3270* affects the biofilm formation of *E. coli* and *M. smegmatis*. The BFI increased by more than double for *rv3270*-harbouring cells of *M. smegmatis* and *E. coli* as compared to the control without *rv3270*. Furthermore, to confirm the role of Rv3270 in influencing multi-drug efflux and biofilm formation, an efflux pump blocker, reserpine, was used. As reported previously, reserpine directly interacts with the efflux pump proteins by acting as a promising inhibitor [52, 53]. Reserpine not only had an inhibitory effect on antibiotic efflux but also affects the biofilm-forming ability of the cells harbouring *rv3270*.

Initially *E. coli* was used as a model organism to study the activity of Rv3270 as *E. coli* is fast growing, and is easy to culture and genetically manipulate, but the cell surface architecture varied widely between *E. coli* and *M. tuberculosis* [47]. *E. coli* is a Gram-negative bacterium while the highly lipid-rich *M. tuberculosis* is acid-fast [1, 48]. Since Rv3270 is a membrane-bound protein, changes in the cell surface composition would often lead to doubtful expression of the protein. Therefore, *M. smegmatis,* a closer model organism, was considered. *M. smegmatis* is a non-pathogenic, fast-growing micro-organism which has similar cell membrane architecture to *M. tuberculosis*. Hence, over-expression of Rv3270 was also studied in *M. smegmatis*. It was of note that the effect of the mycobacterial efflux pump was comparable to that observed in the Gram-negative species *E. coli*.

## Conclusion

Rv3270, a P-type ATPase of *M. tuberculosis*, is known for its role in the transport of divalent cations such as manganese and zinc, maintaining metal homeostasis inside the cell and preventing metal toxicity. The present study revealed that similar to an ABC superfamily transporter Rv1273c which was observed to be a multi-drug transporter that utilizes ATP hydrolysis to efflux antibiotics, Rv3270 also contributes to providing low- to moderate-level tolerance towards various structurally unrelated classes of antibiotics, which are often used as first- and second-line anti-tubercular drugs, by enhancing the efflux pump activity with a preference towards fluoroquinolones and beta-lactams [54]. Zinc positively facilitates the activity of Rv3270 in enhancing the efflux of antibiotics. Therefore, it can be concluded that Rv3270 might play a role in providing drug–metal cross-resistance.

## Supplementary Data

Supplementary material 1
