# Supplementary material for: P-type ATPase zinc transporter Rv3270 of Mycobacterium tuberculosis enhances multi-drug efflux activity
Source: Microbiology (Reading). 2024 Feb 19;170(2):001441. doi: 10.1099/mic.0.001441 (PMC10924464; doi:10.1099/mic.0.001441)
Supplement: Supplementary material 1 [file mic-170-1441-s001.pdf]

Supplementary section of manuscript titled: **P-type ATPase zinc transporter Rv3270 of *Mycobacterium tuberculosis* enhances multi-drug efflux activity**

Table S1: Minimum Inhibitory Concentrations of various unrelated classes of antibiotics against, *M. smegmatis* cells harboring pMIND vector (control of *M. smegmatis* cells) and Rv3270 (pM3270) in presence and absence of 30ppm Zn^{2+}.

Antibiotics	Minimum Inhibitory Concentration (MIC) mg L^{-1}			
			Zn^{2+} (30ppm)	
	M. smegmatis MC^2155	*M. smegmatis* MC^2155/pM3270	*M. smegmatis* MC^2 155	*M. smegmatis* MC^2155/pM3270
Norfloxacin	2	8	2	8
Ofloxacin	0.5	2	0.5	2
Sparfloxacin	2	8	2	8
Ampicillin	32	128	32	128
Oxacillin	16	64	16	64
Amikacin	4	8	4	8
Isoniazid	32	256	32	256
Levofloxacin	2	2	2	8
Apramycin	8	8	8	16
Rifampicin	1	1	1	2

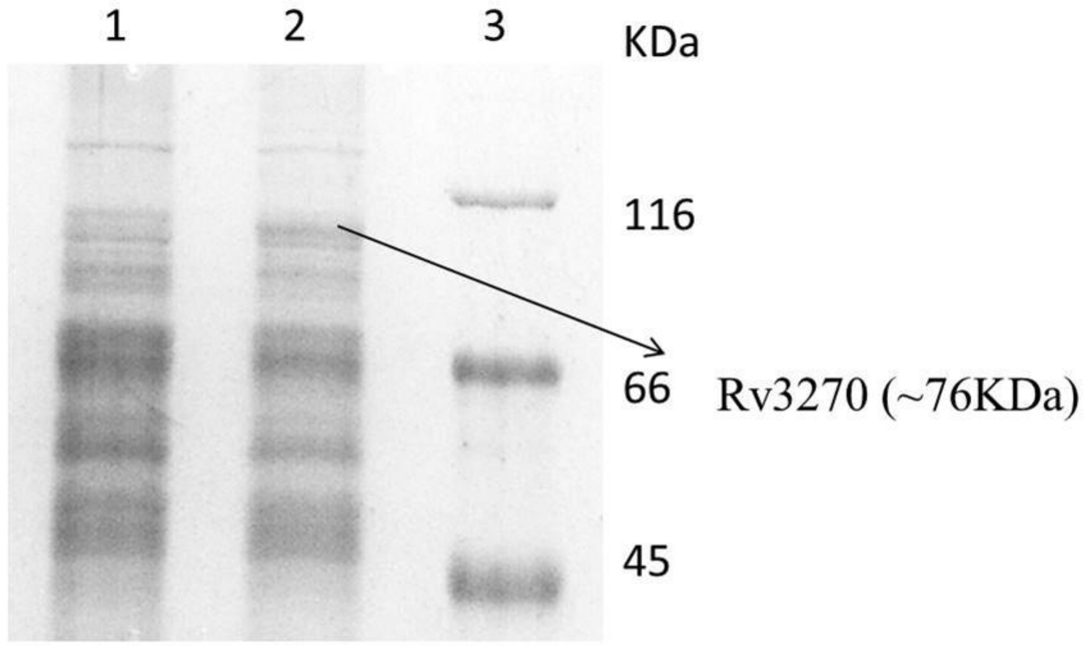

Lane 1: Uninduced Rv3270-pMIND
Lane2: Induced Rv3270-pMIND (20 ng/µL)
Lane 3: Protein Marker

Figure S1: SDS-PAGE image of solubilized membrane fraction of *M. smegmatis* MC^2/155 cells harboring pM3270 upon inducing with 20 ng/µL tetracycline.

Using the overnight culture (0.1%) 10 mL 7H9 broth was inoculated and incubated at 37ºC with a shaking speed of 150 rpm till the culture reached an OD_{600} of 0.2. The expression of the gene and subsequent protein production was induced with 20 ng/µL of tetracycline and incubated for 20-24 hrs. The cells were spun down at 10000 x g for 10 mins at 4ºC in Eppendorf 5810R. The supernatant was discarded and the pellet was washed with 1 mL 10mM Tris-Cl buffer (pH-7.5) and resuspended in the same buffer. The protease inhibitor, phenylmethylsulfonyl fluoride (PMSF), was added at a final concentration of 1 mM to the cell suspension and it was sonicated with five pulses of 60 sec each in a Corning CoolRack M6 placed in ice, followed by centrifugation at 16,000 x g for 10 mins. The supernatant fraction was collected and further centrifuged at 4ºC for 1hr at 20,000 x g. The supernatant was conserved and the pellet fraction was further resuspened in 100 µL of Tris-Cl buffer (pH- 7.5). The pellet fraction was treated with 2% sarcosyl (sodium lauroyl sarcosinate) (w/v), mixed thoroughly and incubated at 37°C on a thermomixer (Eppendorf, Hamburg, Germany) with shaking for 1 hr to solubilize the membrane proteins. The sample was further centrifuged 20,000 x g for 1 hr at 4°C and the

supernatant was recovered. The proteins in recovered supernatant were analyzed through sodium dodecyl sulphate polyacrylamide gel electrophoresis (SDS-PAGE) (12% acrylamide).

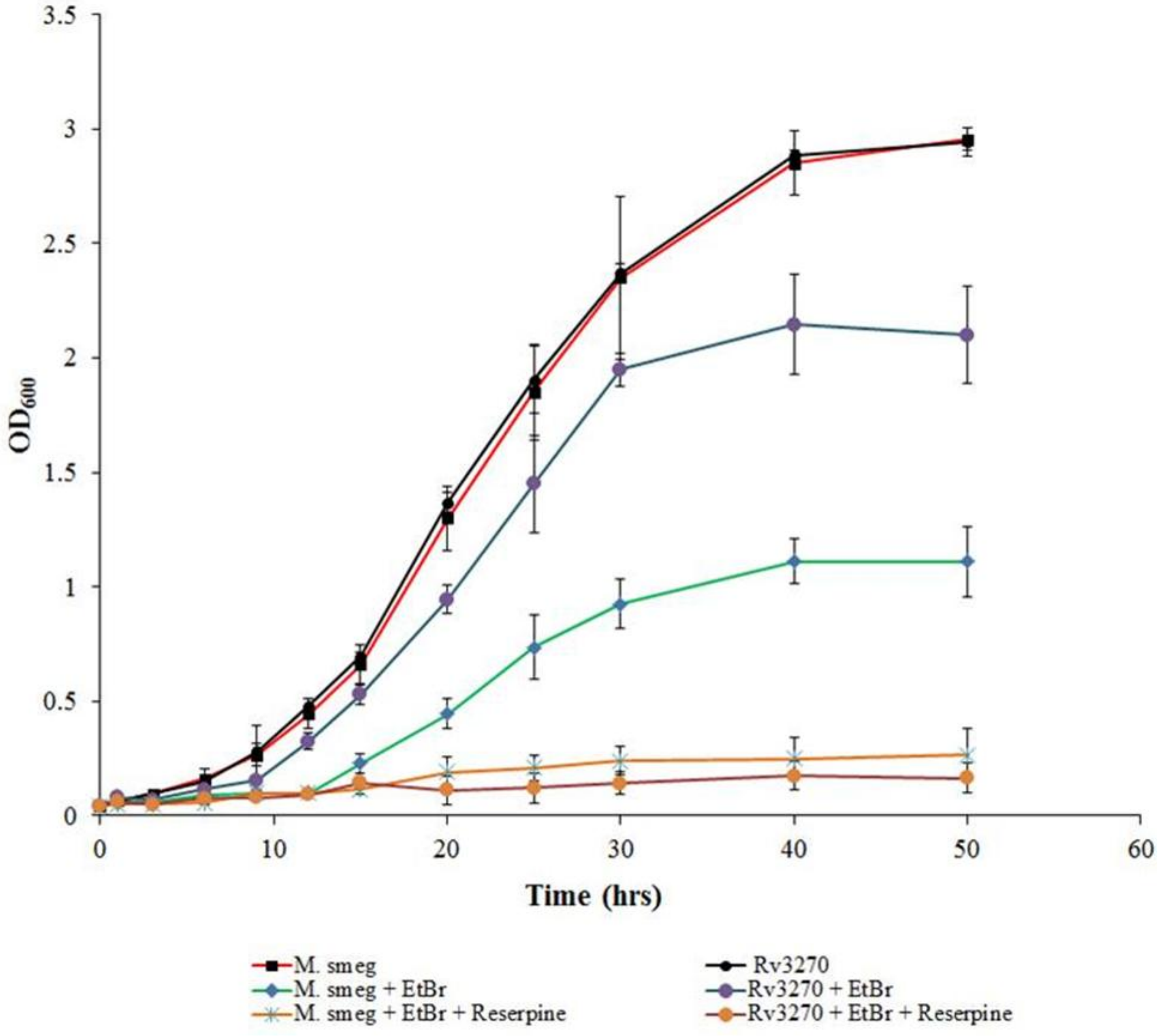

Figure S2: Growth curve analysis of 20 ng/µL tetracycline induced *M. smegmatis* cells over-expressing Rv3270 in presence of 1.5µM EtBr and 256µg mL $^{-}$1 of Reserpine. Red and black lines denote empty vector control *M. smegmatis* cells and *M. smegmatis* cells harboring Rv3270 growth curves. The green and purple lines represent the growth patterns of empty vector control *M. smegmatis* cells and *M. smegmatis* cells harboring Rv3270 in presence of EtBr and red and orange lines denotes the growth patterns of empty vector control *M. smegmatis* cells and *M. smegmatis* cells harboring Rv3270 in presence of EtBr and reserpine.

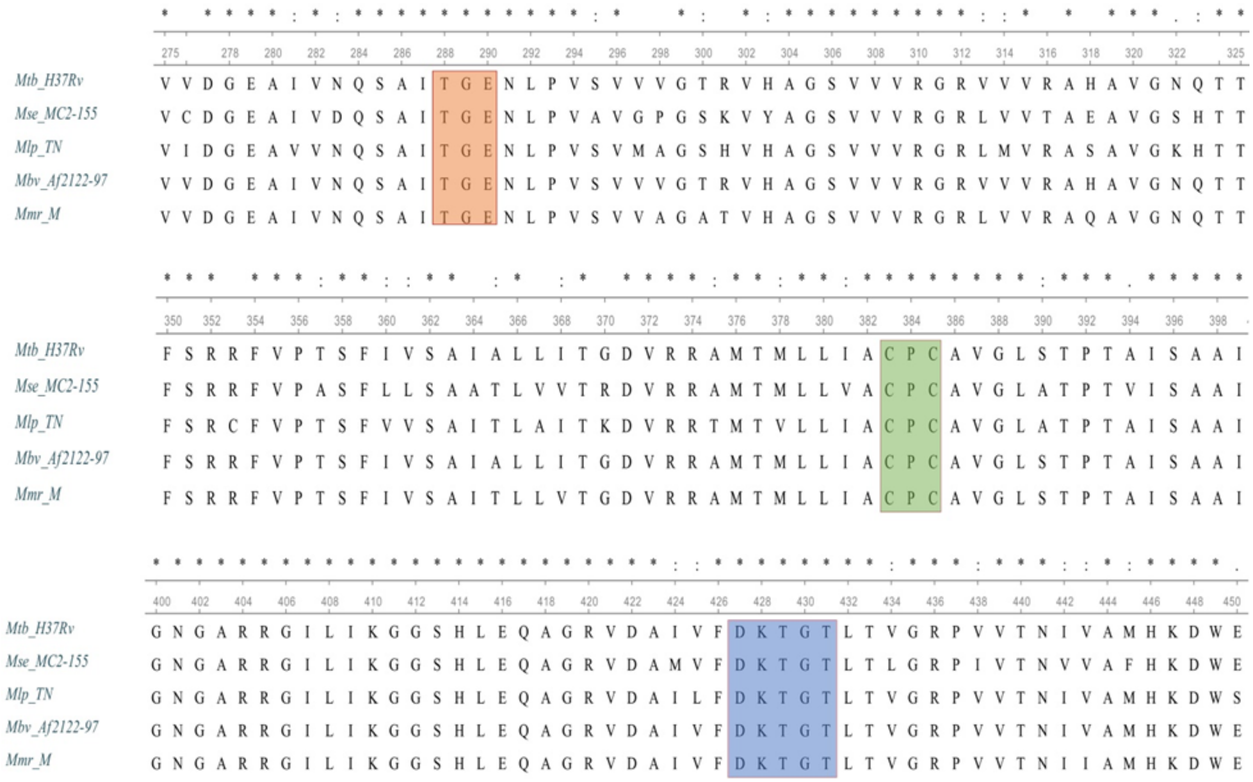

Figure S3: Partial alignment of Rv3270 and its homologous protein sequence correspond to the signature motif of ATP binding domain, transmembrane metal binding site and catalytic phosphorylation domain; from top to bottom respectively. Sequences are from *M. tuberculosis* (Mtb_H37Rv), *M. smegmatis* (Mse_MC2-155), *M. leprae* (Mlp_TN), *M. bovis* (Mbv_Af2122-97) and *M. marinum* (Mmr_M).

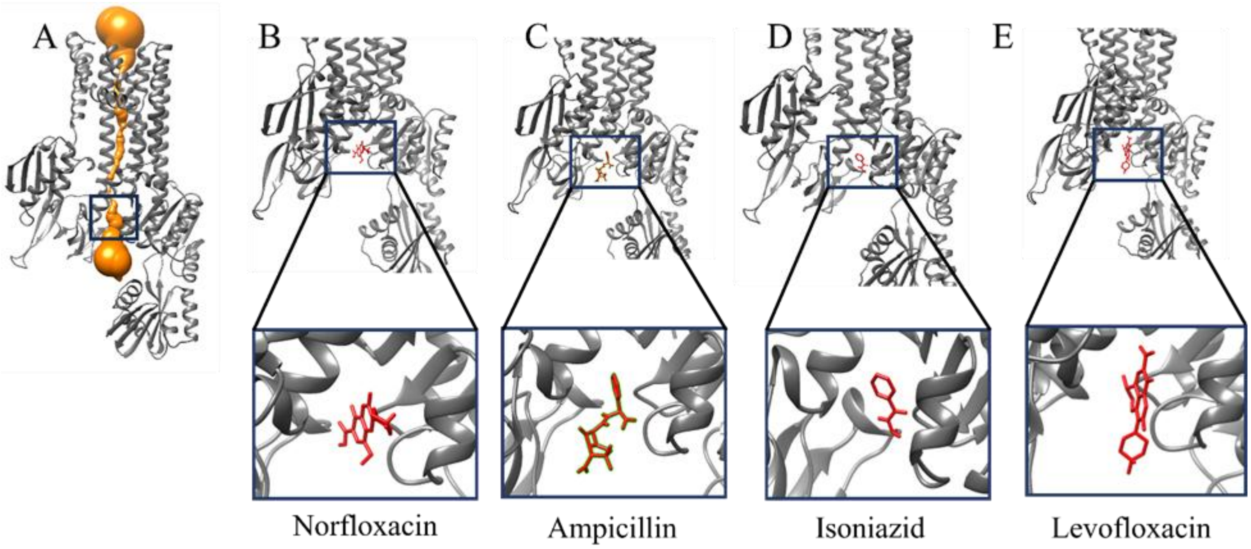

Figure S4: Predicted channel and antibiotic binding site relative to Rv3270 model structure. (A). Predicted channel in Rv3270 model with MOLE2 server. (B-E). Relative binding site of norfloxacin, ampicillin, Isoniazid and levofloxacin in Rv3270 predicted structure respectively. Binding site of antibiotics in 'A' is highlighted in rectangular box
